# Cardiopulmonary exercise test results do not change over two sequential days in patients with chronic fatigue syndrome

**DOI:** 10.3389/fphys.2026.1816082

**Published:** 2026-05-13

**Authors:** Donna M. Mancini, Dane B. Cook, Danielle L. Brunjes, Tiffany Soto, Michelle Blate, Patrick Quan, Tadahiro Yamazaki, Anna Norweg, Benjamin H. Natelson

**Affiliations:** 1 Departments of Cardiology, Icahn School of Medicine at Mount Sinai, New York, NY, United States; 2 Population Health Science and Policy, Icahn School of Medicine at Mount Sinai, New York, NY, United States; 3 Department of Kinesiology, University of Wisconsin, Madison, WI, United States; 4 Neurology, Icahn School of Medicine at Mount Sinai, New York, NY, United States

**Keywords:** cardiopulmonary exercise, chronic fatigue disorder, fatigue, peak VO_2_, post-exertional malaise

## Abstract

**Background:**

Two consecutive cardiopulmonary exercise tests (CPETs) performed 24 hours apart are increasingly used to determine post-exertional malaise (PEM) and disability in patients with myalgic encephalomyelitis/chronic fatigue syndrome (ME/CFS). Declines in functional capacity on Day 2 reflect impaired recovery and PEM. However, reports have variably described a reduction in peak oxygen consumption (VO_2_) and/or VO_2_ at the anaerobic ventilatory threshold (VT). Given the inconsistent findings, we sought to replicate the studies by performing sequential 2-day CPETs in ME/CFS and age- and sex-matched sedentary controls.

**Methods:**

Accordingly, maximal bicycle ergometer CPETs were performed on two consecutive days in 58 patients with ME/CFS (mean age 38.6 ± 9.6 years, Body Mass Index (BMI) 24.1 ± 3.3 kg/m^2^, 11 men and 47 women) and 25 age-matched sedentary control (CON) subjects (age 38.2 ± 9.9 years, BMI 24.2 ± 3.4 kg/m^2^, 5 men and 20 women). Peak VO_2_ was reported as the highest 30-sec average; VT was selected as the nadir of the VE/VO_2_ and P_ET_CO_2_ curves, and VE/VCO_2_ as the slope throughout exercise.

**Findings:**

For ME/CFS and CON subjects, there were no significant changes in Peak VO_2_ between Day 1 and Day 2 studies (ME/CFS Day 1, 22.3 ± 5.4; Day 2, 22.5 ± 5.4 mL·kg^−1^·min^−1^; CON Day 1, 23.4 ± 3.5; Day 2, 22.8 ± 3.6 mL·kg^−1^·min^−1^; NS). Similarly, VO_2_VT and VE/VCO_2_ slopes were not significantly different between the ME/CFS patients and CON, and on Day 2, they did not show any differences within or between groups. Peak heart rate was significantly higher in CON versus ME/CFS. The level of perceived exertion was significantly greater at all levels of exercise on the Day 1 and Day 2 tests for ME/CFS patients versus CON.

**Interpretation:**

Our data indicate that 2-day CPET provides exercise-related results that are the same in ME/CFS patients and CON subjects. ME/CFS patients have a greater perception of exertion throughout exercise and a lower maximum heart rate than CON. The data do not support using the 2-day CPET protocol to define PEM or disability.

## Introduction

Myalgic encephalomyelitis/chronic fatigue syndrome (ME/CFS) is a medically unexplained illness characterized by at least 6 months of unexplained fatigue, severe enough to produce a substantial reduction in activity. In addition to fatigue, patients report symptoms including dyspnea, mental fog, and worsening fatigue following minor physical or mental efforts [post-exertional malaise (PEM)] (Fukuda et al., 1994). This PEM is common in ME/CFS and is a key characteristic of this illness, such that even minimal amounts of exertion can trigger subsequent intensification of illness symptoms.

There have been several reports indicating that ME/CFS patients cannot maintain normal exercise-related cardiopulmonary function on the second day of two sequential cardiopulmonary exercise tests (CPETs) (VanNess et al., 2007; VanNess et al., 2010; Vermeulen et al., 2010; Keller et al., 2014; Hodges et al., 2018; Nelson et al., 2019; Van Campen et al., 2020; Keller et al., 2024). Specifically, these researchers find reduced maximal oxygen consumption (peak VO_2_) and/or earlier onset of the anaerobic threshold (VO_2_VT) compared with values from the first day of testing. This inability to sustain these exercise parameters (VanNess et al., 2007; VanNess et al., 2010; Vermeulen et al., 2010; Keller et al., 2014; Hodges et al., 2018; Nelson et al., 2019; Van Campen et al., 2020; Keller et al., 2024) has been used to infer impaired recovery capacity or PEM, and some disability attorneys are using this protocol to provide objective evidence of PEM and disability.

Several small single-center studies have been published showing variability in the level of reproducibility of peak VO_2_ and VO_2_VT on the second test, with changes in peak VO_2_ ranging from +5.3% to −14% and in VO_2_VT of +6.1% to −27% (VanNess et al., 2007; VanNess et al., 2010; Vermeulen et al., 2010; Keller et al., 2014; Hodges et al., 2018; Nelson et al., 2019; Van Campen et al., 2020; Keller et al., 2024). Some investigators (Nelson et al., 2019) have attributed the inconsistency of the data to differences in the patients’ exercise intensity, i.e., failing to achieve maximal exercise on Day 2. Many but not all studies lacked a comparable control group, which the investigators of one paper (Keller et al., 2014) contended was not necessary because neither of these variables decreases on repeat CPET in healthy people. One large recent multicenter study by Keller et al (Keller et al., 2024). included 84 patients with ME/CFS and 71 sedentary control subjects and showed a 5.3% decrease in peak VO_2_ and a 6.8% decline in VO_2_VT in ME/CFS on Day 2 testing, with minimal decline in these parameters for the sedentary controls. Also, a study from Norway showed that arterial lactate on Day 2 CPET was increased at the VO_2_VT in comparison with Day 1 CPET, whereas it was decreased in healthy control subjects (Lien et al., 2019), suggestive of a metabolic abnormality occurring in ME/CFS. Another study showed that after Day 2 CPET, female patients tended to show progressive declines in peak exercise workload that paralleled initial illness severity (Van Campen et al., 2020). Vermeulen and colleagues reported reduced peak oxygen consumption of 1.4 mL·kg^−1^·min^−1^ in ME/CFS on the second compared to the first exercise test but found no differences in outcomes at VT or peak work rate (Vermeulen et al., 2010).

Given the variability of findings by different investigators, we sought to replicate the studies by performing sequential 2-day CPETs in ME/CFS and age- and sex-matched sedentary controls. We hypothesized that many ME/CFS patients would not show declines in CPET and yet still experience PEM.

## Methods

### Patient population

Forty-seven women and 11 men with ME/CFS were included in the study. They all met both the 1994 case definition of ME/CFS, modified to require endorsement of post-exertional malaise (**Table 1**). Thus, they all reported new onset of fatigue, lasting at least 6 months, producing at least a substantial reduction in activity in any of the following spheres: personal, social, occupational, or educational (at least a 3 on a 0–5 Likert scale, where 0 was none, 3 substantial, 4 severe, and 5 was very severe). To fulfill criteria for the diagnosis, patients also had to report at least a substantial burden (same 0–5 Likert scales) on 4 of the following symptoms (sore throat, tender cervical or axillary nodes, headache, myalgia, arthralgia, problems with attention or concentration, and unrefreshing sleep). They were also required to report at least a substantial burden with post-exertional malaise with a VAS of at least 3.

**Table 1:** Demographic data.

Characteristics	ME/CFS	CON
N	58	25
Age (years)	38.6 ± 9.6	38.2 ± 9.9
Gender	11 M and 47 F	5 M and 20 W
Height (in.)	65.2 ± 3.2	65.6 ± 3.7
Weight (lb)	145.8 ± 26.4	148.9 ± 29.4
BMI	24.1 ± 3.3	24.2 ± 3.4
Godin scale	27.7 ± 35.5	17.6 ± 14.4

ME/CFS, myalgic encephalomyelitis/chronic fatigue syndrome; CON, matched sedentary control subjects.

The exclusion criteria were patients with a medical cause for their fatigue, those taking medications that would dampen cardiac response to exercise, or those with one of the following psychiatric conditions: psychotic illness; bipolar disorder; history of anorexia or bulimia within 5 years of intake or history of alcohol or drug abuse within 2 years of intake; or current major depressive disorder. Patients with Ehlers–Danlos syndrome were also excluded. The average age of the ME/CFS patients was 38.6 ± 9.6 years, and they had a BMI of 24.1 ± 3.3 kg/m^2^.

Twenty women and five men constituted our healthy control sample (CON). These subjects reported being sedentary, not exercising regularly, having no medical problems, and not taking any medications other than birth control pills. CONs were comparable to ME/CFS patients for age, gender, conditioning status (Godin Activity Scale), and BMI. Sixteen of the 20 control subjects were employed, and all had sedentary desk jobs. The average age of the sedentary controls was 38.2 ± 9.9 years, and they had a BMI of 24.2 ± 3.4 kg/m^2^. All ME/CFS and CON completed the Godin Activity Scale. The weekly leisure time activity score was 27.7 ± 35.5 in the ME/CFS group and 17.6 ± 14.4 (p = 0.217) in CON, indicating comparable levels of activity.

The study was approved by the Mount Sinai Institutional Review Board. All participants provided signed, written informed consent. The first participant was enrolled on August 26, 2021, and the final participant on June 17, 2025.

### Cardiopulmonary exercise tests

Patients reported to the exercise laboratory in the fasting state. Medications affecting heart rate or blood pressure were discontinued 1 week prior to exercise and resumed following the second exercise test. Patients were connected to an EKG, pulse oximeter, and BP cuff and seated on a bicycle ergometer (Lode, Groningen, Netherlands).

Using a disposable mouthpiece, the patients breathed into a metabolic cart (MCGDiagnostics, St Paul, Minnesota Ultima O_2_). Resting data were collected for 2 min, and then incremental bicycle exercise was begun at 0 W, increasing by 25 W every 2 min to exhaustion. VO_2_ consumption, VCO_2_ production, respiratory frequency, minute ventilation, end tidal CO_2_, and O_2_ were recorded continuously. O_2_ saturation was also recorded. Blood pressure and perceived exertion using the Borg scale were obtained at each workload and at peak exercise. The reason for terminating the exercise was recorded. The cardiopulmonary exercise test was repeated in 24 hours.

Peak VO_2_ was defined as the highest 30-sec average of oxygen consumption and was normalized by the predicted VO_2_ (Wasserman equation) to derive a % predicted value. For the lactate threshold or gas exchange threshold (GET), we reported it as the ventilatory threshold (VO_2VT_). VO_2VT_ was determined independently by Drs Mancini and Brunjes, using a concordance of the following criteria: 1) analysis of the nadir of the V_E_/VO_2_ and V_E_/VCO_2_ using the breath by breath data, 2) the V slope method using the anaerobic threshold plot generated by the metabolic cart, and 3) identification of the nadir of the end tidal pressure of oxygen (PetO_2_). If the VO_2VT_ determined by Drs Mancini and Brunjes differed, Dr Cook reviewed the tests.

It was identified as the point at which the ventilatory equivalent for O_2_ was minimal, followed by a progressive increase. Ventilation (VE) carbon dioxide (VCO_2_) slope was assessed by correlation of VE and VCO_2_ throughout the exercise. Normal VE/VCO_2_ slope is <30. A maximal test was determined according to the criteria of the American College of Sports Medicine (2022) when at least two of the following were met: 1) a plateau in VO_2_ with increasing work rate, 2) maximal heart rate (HR) ≥85% predicted, 3) Respiratory exchange ratio (RER) ≥ 1.1, 4) lactate concentration >8 mmol, and 5) rating of perceived exertion (RPE) ≥17. In these studies, lactate was not measured, so only the remaining criteria were used to define a maximum test here. Of these criteria, nearly every subject achieving maximum did so via RER and RPE.

### Data and statistical analysis

All continuous variables are presented as mean ± standard deviation. Variables were compared by non-paired t-testing, assuming equal variance and reported as significant if two-tailed p < 0.05. Categorical variables were summarized as frequencies and percentages. Levene’s test statistic for homogeneity was used to test equal variances between groups. Repeated-measures ANOVA was used.

## Results

### Respiratory gas analysis

The results of those ME/CFS patients and controls who achieved maximal effort on both tests are shown in **Table 2**. Five ME/CFS and six sedentary controls did not meet the American College of Sports Medicine (ACSM) criteria. For the ME/CFS patients, nearly every subject achieving max did so via RER and RPE. Of the ME/CFS patients, 21 and 18 patients did not achieve >85% predicted maximal HR on Day 1 and Day 2 of testing, versus only two sedentary controls on Day 1 and Day 2. Nine ME/CFS subjects achieved HR max below 80% on Day 1 and Day 2, versus no control subject on Day 1 and 1 on Day 2.

**Table 2:** ME/CFS and sedentary controls with maximal Day 1 and Day 2 tests.

	ME/CFS test 1		ME/CFS test 2		CON test 1		CON test 2	
Parameter	Rest	Peak	Rest	Peak	Rest	Peak	Rest	Peak
HR (bpm)	81.1 ± 13.5	156.7 ± 18.6	82.6 ± 13.7	155.4 ± 17.5	84.5 ± 11.7	166.3 ± 11.1*	82.6 ± 12.5	168.1 ± 11.7**
Predicted HR (%)		86 ± 10		85 ± 9		90 ± 8		91 ± 8*
mean arterial blood pressure (mBP) (mmHg)	87.1 ± 8.5	102.9 ± 11.7	86.9 ± 9.0	101.8 ± 11.0	89.5 ± 6.3	106.1 ± 7.7	89.2 ± 5.8	106.7 ± 7.7
VO_2_ (mL·kg^−1^·min^−1^)	4.2 ± 0.8	22.3 ± 5.4	4.4 ± 0.8	22.5 ± 5.4	3.95 ± 0.82	23.4 ± 3.5	4.2 ± 0.7	22.8 ± 3.6
Predicted VO_2_ (%)		78·4 ± 18·9		79 ± 19		80 ± 11		76 ± 9
RER	0·91 ± 0.11	1.21 ± 0.08	0.90 ± 0.12	1.21 ± 0·09	0.92 ± 0.14	1.24 ± 0.09	0.92 ± 0.10	1.24 ± 0.08
Respiratory rate (RR) (n)	15 ± 5	32 ± 7	15 ± 5	32 ± 7	15 ± 5	35 ± 8	16 ± 4	35 ± 7
P_ET_ CO_2_ (mmHg)	34.2 ± 5	37.4 ± 4.9	34.3 ± 6.3	37.3 ± 5.6	35.5 ± 5.0	39.4 ± 4.9	35.2 ± 4.6	38.5 ± 3.4
VO_2_VT (mL·kg^−1^·min^−1^)		12.4 ± 3.3		12.9 ± 3.4		12.4 ± 2.5		12.4 ± 3.0
VE/VCO_2_		29.1 ± 5.9		29.2 ± 5.7		27.8 ± 3.8		28.2 ± 3.8
Borg scale		18.1 ± 1.2		18.8 ± 1.0		16.5 ± 2.5*		17.3 ± 2.4*
OUES		1626 ± 449		1631 ± 429		1753 ± 436		1764 ± 445
VO_2_ pulse (mL/beat)		9.3 ± 2.5		9.5 ± 2.5		9.7 ± 2.7		9.4 ± 2.5
Peak workload (W)		127 ± 35		127 ± 34		130 ± 28		132 ± 27

ME/CFS, myalgic encephalomyelitis/chronic fatigue syndrome; CON, matched sedentary control subjects; HR, heart rate; OUES, oxygen uptake efficiency slope. * p < 0.05 controls versus ME/CFS; **p < 0.01 controls versus ME/CFS.

**Figure 1:**
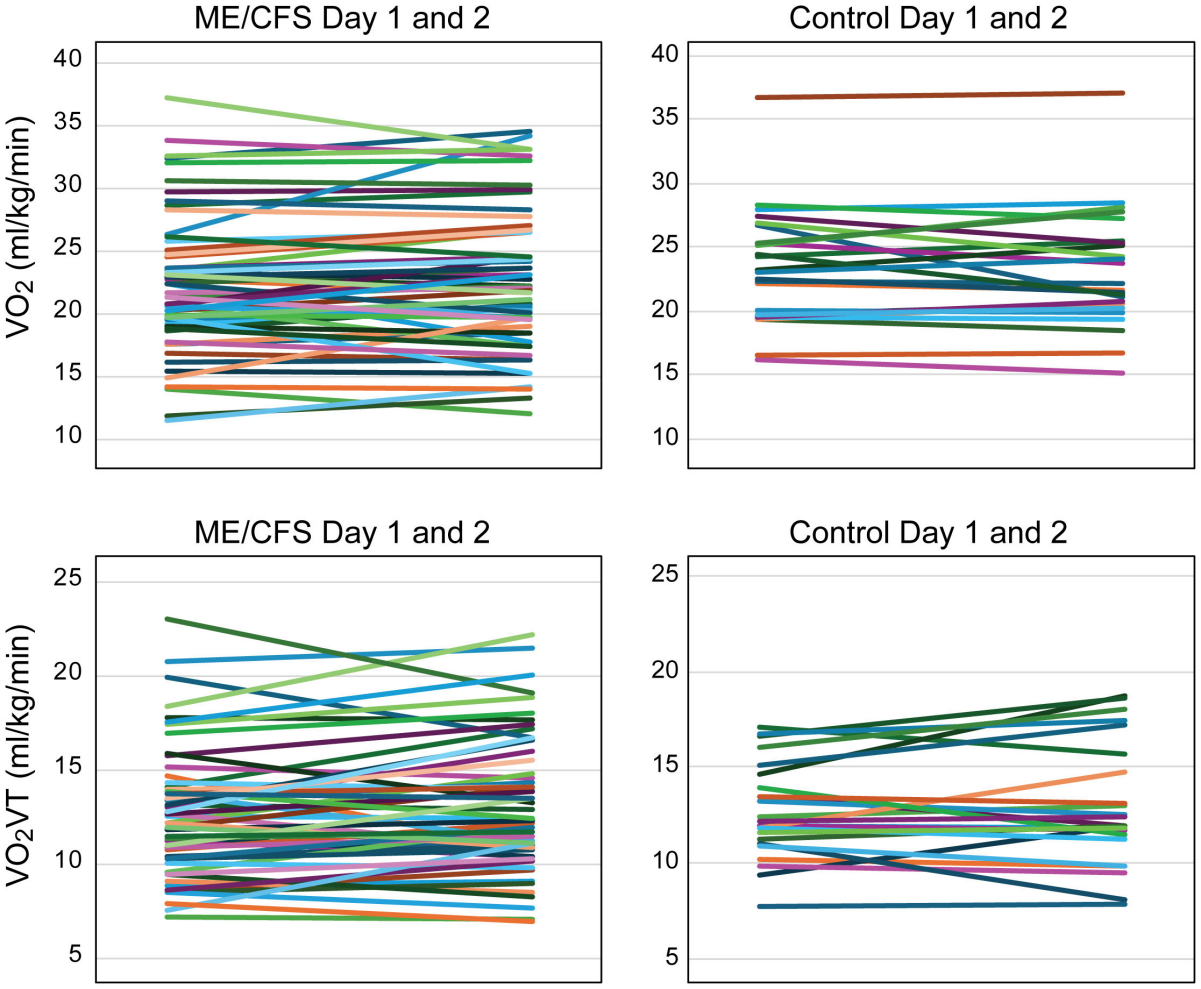
Peak VO_2_ on Day 1 and Day 2 for ME/CFS **(a)** and control patients **(b)**. VO_2_VT on Day 1 and Day 2 for ME/CFS **(c)** and control patients **(d)**. ME/CFS, myalgic encephalomyelitis/chronic fatigue syndrome.

For the ME/CFS and CON subjects, there were no significant changes in peak VO_2_ between Day 1 and Day 2 studies (ME/CFS Day 1, 22.3 ± 5.4; Day 2, 22.5 ± 5.4 mL·kg^−1^·min^−1^; CON Day 1, 23.4 ± 3.5; Day 2, 22.8 ± 3.6 mL·kg^−1^·min^−1^; NS). Levene’s test statistic for homogeneity of variance was 0.621 with a p-value of 0.433, confirming equal variances. Maximum heart rate was statistically higher on Day 1 and Day 2 in controls versus ME/CFS subjects (ME/CFS Day 1, 157 ± 19; Day 2, 155 ± 18 bpm; CON Day 1, 166 ± 11; Day 2, 168 ± 12 bpm; p < 0.05). Similarly, VO_2_VT was not significantly different between the ME/CFS patients and sedentary controls (ME/CFS Day 1, 12.4 ± 3.3; Day 2, 12.9 ± 3.4 mL·kg^−1^·min^−1^; CON Day 1, 12.4 ± 2.5; Day 2, 12.4 ± 3.0 mL·kg^−1^·min^−1^) (**Figures 1A–D**). There were no statistical differences in the workload at the VT threshold on Day 1 or Day 2 for the CFS patients (Day 1, 58 ± 23; Day 2, 62 ± 26 W; p = 0.07) or exercise duration (Day 1, 666 ± 170; Day 2, 679 ± 161 sec; p = 0.24). In order to increase our sample size, we used an RER ≥ 1.05 to define a maximum test. Using this approach, only two patients had lower RER values (one patient on Day 1 with RER of 1.03 and one on Day 2 with RER = 1.02). Adding the additional patients with RER between 1.05 and 1.1 did not change the results: peak VO_2_ was not different on Day 1 or Day 2 (Day 1 peak VO_2_, 22.3 ± 5.4; Day 2, 22.6 ± 5.6 mL·kg^−1^·min^−1^; p = NS).

Thirteen of the 58 ME/CFS (22%) had a 1 mL·kg^−1^·min^−1^ or greater decline in peak VO_2_ on Day 2. For these 13 ME/CFS patients, VO_2_ declined approximately 10% from 21.8 ± 6.3 to 19.6 ± 6.2 mL·kg^−1^·min^−1^ (p < 0.01). There tended to be more men (n = 4; p = 0.08). Similarly, eight of 25 sedentary controls (33%) had a 1 mL·kg^−1^·min^−1^ or greater decline in peak VO_2_ on Day 2, with peak VO_2_ declining approximately 10% from 25.0 ± 4.1 to 22.5 ± 3.7 mL·kg^−1^·min^−1^ (p = NS CON vs ME/CFS).

**Figure 2:**
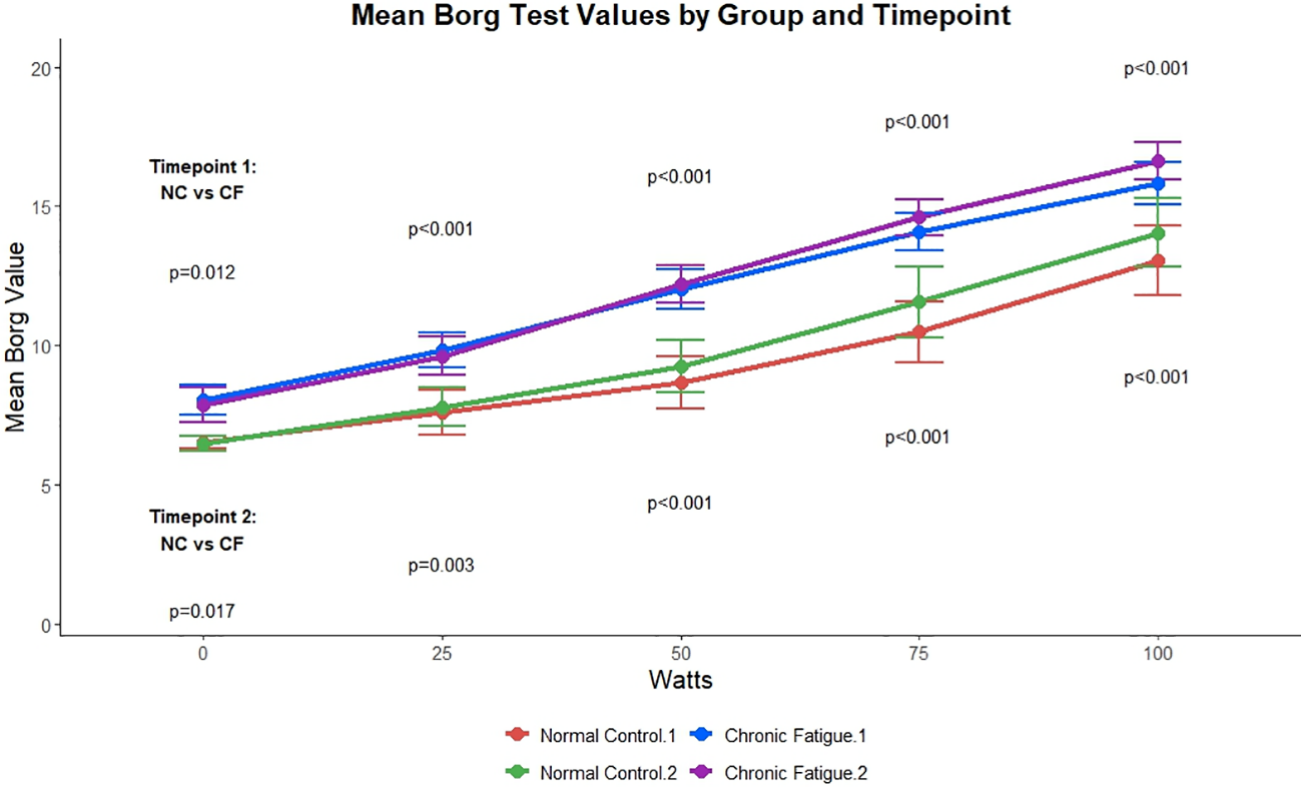
Borg scale of perceived fatigue for ME/CFS and sedentary controls for submaximal and maximal workloads. ME/CFS, myalgic encephalomyelitis/chronic fatigue syndrome.

The number of subjects with VO_2_ below 20 mL·kg^−1^·min^−1^ did not differ between ME/CFS and CON (p = 0.11 ME/CFS vs CON). Perceived effort, as quantified by the Borg scale, was similar on Day 1 and Day 2 in ME/CFS but was greater than that of sedentary controls on both days (**Figure 2**). Perceived maximal effort was also significantly lower in the sedentary controls (**Table 3**).

**Table 3:** Borg scale during exercise in ME/CFS and sedentary controls.

	ME/CFS		CON	
Workload (W)	Day 1 Borg	Day 2 Borg	Day 1 Borg	Day 2 Borg
0	8.0 ± 1.9*	7.9 ± 2.3*	6.5 ± 0.5	6.5 ± 0.6
25	9.8 ± 2.3**	9.6 ± 2.5**	7.6 ± 1.9	7.8 ± 1.6
50	12.0 ± 2.7**	12.2 ± 2.5**	8.7 ± 2.2	9.3 ± 2.2
75	14.1 ± 2.5**	14.6 ± 2.3**	10.5 ± 2.5	11.6 ± 3.0
100	15.9 ± 2.6**	16.6 ± 2.3**	13.0 ± 2.9	14.0 ± 2.8
Maximal	18.2 ± 1.5**	18.7 ± 1.2**	16.4 ± 2.8	17.2 ± 2.4

ME/CFS, myalgic encephalomyelitis/chronic fatigue syndrome; CON, matched sedentary control subjects. *p < 0.05 ME/CFS vs CON; ** p < 0.001 ME/CFS vs CON.

## Discussion

In our cohort of patients with ME/CFS and sedentary healthy control subjects, we found no change in peak VO_2_, VO_2_VT, VO_2_ pulse, and VE/VCO_2_ between Day 1 and Day 2 CPETs. ME/CFS and sedentary controls performed similarly; however, perceived effort was greater and maximal heart rate lower in ME/CFS than CON.

Several reports have described a decrease in peak VO_2_ or a decline in VO_2_VT as a marker for PEM in ME/CFS patients (VanNess et al., 2007; VanNess et al., 2010; Vermeulen et al., 2010; Vermeulen et al., 2010; Keller et al., 2014; Hodges et al., 2018; Nelson et al., 2019; Van Campen et al., 2020; Keller et al., 2024). Also of note, the changes across parameters are generally small. The largest cohort by Keller et al (Keller et al., 2024). included 84 patients with ME/CFS and 71 control subjects. These authors reported significant declines in exercise time, VO_2_ (5.3%), VE (7.2%), VO_2_VT (6.7%), VCO_2_, TV, HR, O_2_ pulse, and diastolic BP. ME/CFS patients who met criteria for maximum effort on Day 1 CPET but who did not meet these criteria on Day 2 were included. The number of subjects attaining a RERmax of 1.1 per the ACSM criteria was not reported in that paper; instead, they noted that “for CPET-2, there was a significant decline in [age-adjusted percent heart rate reserve] %HRR adjusted for ME/CFS in the total sample (*p* ≤ 0.01) and matched-pairs (*p* ≤ 0.05) that fell below the threshold of 80%, suggesting that maximum effort was not given during Day 2 CPET” (Keller et al., 2024). This lack of effort on Day 2 testing could explain the apparent decrement in VO_2_max noted. In our cohort, there was no significant change in % predicted max HR on Day 1 or Day 2. It is unlikely that the discrepancy between Keller’s results and ours is due to differences in the clinical criteria used to define ME/CFS, as a CDC multicenter study found that there were no differences in any variable using either the Canadian or Fukuda criteria (Unger et al., 2024).

The lack of decline in exercise performance does not preclude subjective symptoms post-exercise consistent with PEM. Therefore, we monitored symptoms (via ecological momentary assessment on a wrist-mounted computer) across each day for the week prior to CPET and the week following it. These results will be reported in a separate publication, but preliminarily, we can report that ME/CFS patients could be differentiated from HCs across most symptoms and for most post-CPET days.

A lower maximal heart rate was more common in ME/CFS patients, contributing to their exercise intolerance. Chronotropic incompetence (i.e., maximal HR < 80% predicted) was observed in nine ME/CFS versus only one sedentary control subject. Autonomic dysfunction, beta receptor desensitization, deconditioning, and background medications could be factors resulting in the lower achieved maximal heart rate.

In contrast to Keller (Keller et al., 2024), we were able to obtain maximal effort as determined by RER on Day 2 in all but five patients. Lowering the RER criterion to ≥ 1.05 allowed us to capture every ME/CFS patient except one with no change in the results. In 2020, Davenport et al (Davenport et al., 2020). also reported on 51 women with ME/CFS and 10 sedentary female patients matched for age and body size. The primary objective of the study was to demonstrate the reproducibility of CPET parameters. It did demonstrate reproducibility, and although the peak VO_2_ was higher in the control subjects than ME/CFS on Day 1 and Day 2, the decrement in peak VO_2_ on Day 2 was comparable for both groups. Van Campen studied 82 female patients with ME/CFS with 2-day CPETs and stratified results by disease severity (Van Campen et al., 2020). There was no control group. For mild, moderate, and severe disease, there were comparable declines in peak VO_2_ on Day 2. Workload at peak exercise declined in all groups on Day 2, with RER and maximum heart rate prone to being lower on Day 2, but did not reach statistical significance.

Our findings are important, as the absence of significant changes in CPET does not support the use of two sequential CPETs to infer either PEM or disability. Exercise scientists who plan to continue using the 2-day CPET protocol should require patients to attain an RER of at least 1.05; if patients do not attain this on Day 2 CPET, that would explain any reduction in peak VO_2_ found.

Interestingly, perceived exertion across the exercise test duration was significantly higher in ME/CFS than in sedentary controls and is consistent with prior work (Cook et al., [[NoYear]]; Barhorst et al., 2020), again suggesting that symptom assessment post-exercise testing may be the most useful method to identify PEM.

## Conclusion

Changes in peak VO_2_ or VO_2_VT on 2-day CPET changes do not appear to be a good marker to determine disability or to identify post-exertional malaise in patients with ME/CFS. The addition of subjective monitoring of symptoms following exercise testing may be a more sensitive and specific marker of PEM.

## Data Availability

The raw data supporting the conclusions of this article will be made available by the authors, without undue reservation.
